# Characteristics and karyotype analysis of a patient with turner syndrome complicated with multiple-site tumors: A case report

**DOI:** 10.1515/biol-2022-0499

**Published:** 2022-11-11

**Authors:** Li Yang, Yu Yang, Yao Qin, Ya-Qin Feng, Li-Ling Xie, Dong-Guang Zhang

**Affiliations:** Department of Endocrinology, Genetic and Metabolism, Nanchang University Affiliated Children’s Hospital/Jiangxi Province Children’s Hospital, Jiangxi Province Clinical Medical Research Center for Children’s Genetic and Metabolic Diseases, No. 1666, Dizihu Avenue, Honggutan District, Nanchang 330006, China

**Keywords:** turner syndrome, abnormal sexual development, adrenal tumor, gonadoblastoma

## Abstract

Turner syndrome (TS) is a congenital chromosomal abnormality that affects approximately 1 in 2,500 people. Both in China and abroad, few studies exist on the incidence of tumors in patients with TS. Most reported cases are complicated with gonadal germ cell tumors, and extragonadal tumors are rare, with the latter not yet being reported in China. Through chromosome karyotype analysis and surgical exploration, a pediatric patent was diagnosed with TS complicated with gonadoblastoma and adrenal neuroblastoma. The patient was short in stature and had a facial deformity. After admission, adrenal computed tomography was conducted, and a right adrenal mass was identified as a neurogenic tumor. After surgical resection and gonadal exploration, the pathological results revealed left gonadoblastoma, right gonadal stromal cell hyperplasia, and ganglion neuroblastoma (mixed type) in the right adrenal gland. Pediatric patients with TS have an increased likelihood of developing neuroblastoma and adrenal-related tumors, and changes in adrenal hormone levels and clinical manifestations are often not obvious when combined with adrenal-related tumors. To avoid missed diagnosis and delayed treatment, screening for adrenal tumors is therefore recommended for patients with TS before the initiation of growth hormone treatment.

## 1 Introduction

Turner syndrome (TS), also known as congenital ovarian hypoplasia, is caused by the loss or structural abnormality of the X chromosome occurring in all or part of the cell during the formation of zygotes. Its incidence is approximately 1 in 2,500 live-born female infants [1]. Generally, TS cases can be divided into 45, XO haplotypes; 46, XX structural abnormalities; and mosaics with or without structural variation. The main clinical manifestations are short stature and gonadal dysplasia. In pediatric patients, TS may be associated with complications like cubitus valgus, a short fourth metacarpal bone, skin pigmented nevus on the face, a webbed neck, or heart and kidney malformations. The cancer risk in patients with TS is not yet clear.

Most reported cases to date are complicated with gonadal germ cell tumors. Extragonadal tumors have only been reported abroad; that is, no such cases have been reported in China. The cases reported in China are mainly complicated with neurogenic tumors. The present study retrospectively analyzed the case of a pediatric patient with TS complicated with gonadoblastoma and adrenal neuroblastoma. The clinical characteristics and therapeutic methods were summarized, and the relevant literature was reviewed to further our understanding of the disease.

## 2 Materials and methods

### 2.1 Subject enrollment

The clinical characteristics, karyotype characteristics, related symptoms, treatment, and results of a patient with TS complicated with gonadoblastoma and adrenal neuroblastoma admitted to Jiangxi Children’s Hospital in July 2021 were retrospectively analyzed. The relevant literature was reviewed to strengthen our understanding of the complications of TS.


**Informed consent:** Informed consent has been obtained from all individuals included in this study.
**Ethical approval:** The research related to human use has been complied with all the relevant national regulations, institutional policies, and in accordance with the tenets of the Helsinki Declaration, and has been approved by the Ethics Committee of Jiangxi Children’s Hospital.

### 2.2 Clinical history

A female patient aged 11 years and 8 months was admitted to the Department of Endocrinology, Genetics, and Metabolism of Jiangxi Children’s Hospital in July 2021 due to short stature of five years’ duration. Her annual growth rate was unknown. No chronic headache, nausea, vomiting, or blurred vision were reported, and there was no obvious delay in motor and intellectual development. The child was the second fetus and second birth of her mother, and the birth was a natural birth at 40 weeks of pregnancy, i.e., at full term. The patient had no history of hypoxia or asphyxia, her birth weight was 2.95 kg, and her initial growth and development were normal. There was no menstrual history. The patient’s father, mother, and brother were in good health. Her parents were not consanguineous, and there was no family history of hereditary disease. The heights of the patient’s father, mother, paternal grandfather, paternal grandmother, maternal grandfather, and maternal grandmother were 155, 150, 165, 145, 160, and 150 cm, respectively.

### 2.3 Physical examination

A physical examination of the patient revealed a temperature of 36.5℃, pulse rate of 98 beats/min, respiratory rate of 20 breaths/min, weight of 29 kg, blood pressure of 110/70 mmHg, height of 119 cm, head circumference of 54 cm, and distance between fingertips of 118 cm. The patient was short and well-proportioned, with normal skin and some moles on her face. Tooth enamel development was normal. The patient’s jaw arch was normal, and the neck was normal. The hairline was normal, and chicken breast, funnel chest, and coastal valgus were absent. The patient had a shielded chest, with a widened breast space. Bilateral breast development was at Tanner stage B2–3, with soft breast nucleus and no nipple retraction. There was no scoliosis. The cardiac sound was strong, and the heart rhythm was steady. There was normal auscultation of both lungs. The abdomen was flat and soft, with no liver or spleen palpable under the ribs. The patient was symmetrical across both sides, with no cubitus valgus, no knee valgus (X leg) or knee varus (O leg), no penetrating palm, and no typical signs of rickets. The patient had a short fifth metacarpal, normal little finger, normal muscle strength, and normal muscular tension. Axillary hair development was at stage A1, and pubic hair development was at Tanner stage PH1.

### 2.4 Laboratory examination

The level of follicle-stimulating hormone (FSH) was 120.55 mIU/mL (with a normal reference range of 1.5–12.8 mIU/mL), and the level of luteinizing hormone (LH) was 18.16 mIU/mL (with a normal reference range of 0.5–12.9 mIU/mL); that is, both were significantly increased. Estradiol was present at <1.8 pg/mL and testosterone at 15.65 ng/dL. The levels of progesterone, cortisol, adrenocorticotropic hormone, 17-hydroxyprogesterone, dehydroepiandrosterone sulfate, androstenedione, insulin-like growth factor-1, and inorganic elements were normal, as were the thyroid function, liver and kidney function, and the results of the routine blood test. A human chorionic gonadotropin (HCG) stimulation test found that testosterone did not increase significantly from 15.26 ng/dL. Chromosome analysis of cultured cells revealed karyotype 45, X[19]/46, X, + mar[21]. Monochrome fluorescence *in situ* hybridization (of XY chromosomes) showed percentages of 40, 5, 52, and 3 for the XY chromosome, XYY chromosome, X chromosome, and Y chromosome, respectively. A growth hormone (GH) stimulation test using arginine and clonidine showed that the level of GH was 5.20 ng/mL. A gonadorelin stimulation test revealed an LH peak value of 169.02 mIU/mL and an FSH peak value of 200 mIU/mL. The results for carcinoembryonic antigen, alpha-fetoprotein, HCG, serum transferrin, and ferritin were normal. Sex-determining region Y gene fragment analysis detected a stripe matching the size of the destination fragment.

### 2.5 Imaging examination

**Figure 1:**
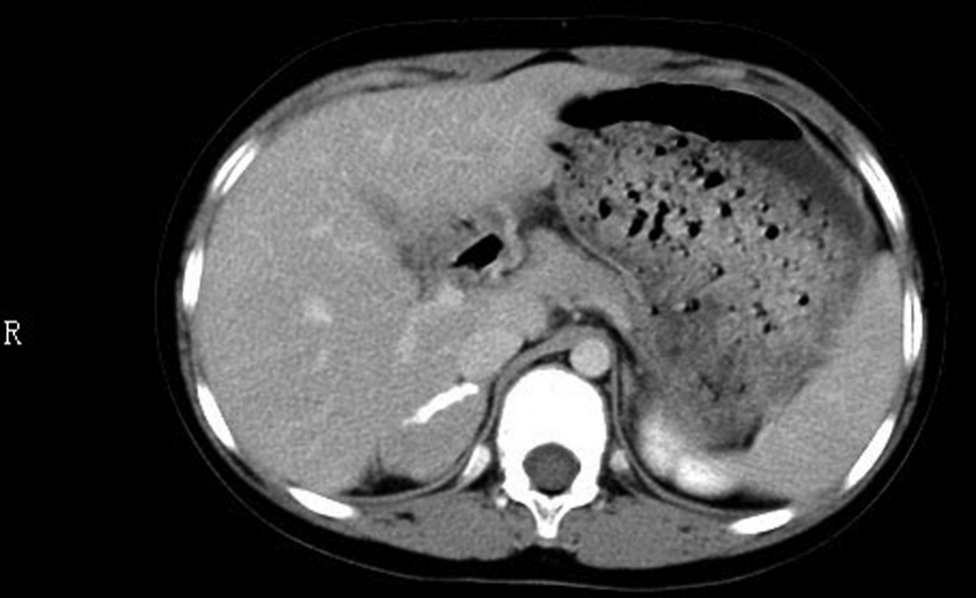
Enhanced CT scan of the patient’s abdomen showed a mass of 139.7 mm × 17.6 mm × 20.3 mm in the right adrenal gland.

An abdominal ultrasound found a hypoechoic mass of approximately 38 mm × 30 mm × 28 mm in the right adrenal gland, which was considered to be a neurogenic tumor. The liver, pancreas, spleen, bilateral kidneys, and left adrenal gland were normal. A two-dimensional ultrasound of the uterus and ovaries revealed a naïve uterus of approximately 26 mm × 12 mm × 6 mm (including the cervix body); the bilateral ovaries were not explored. Adrenal computed tomography (CT) showed that the right adrenal gland was heterogeneous, with blotchy and patchy calcification at the margin. Enhanced scanning showed significant enhancement in the parenchyma, which was 139.7 mm × 17.6 mm × 20.3 mm in size (Figure 1). Neurogenic tumors were the most likely explanation. Electrocardiogram, echocardiography, anteroposterior and lateral spinal radiography, and cranial magnetic resonance imaging (MRI) were normal. An MRI scan of the pituitary gland showed no obvious abnormalities with the cisterna magna. Chest CT showed local pleural thickening on the right side.

### 2.6 Diagnosis and treatment

**Figure 2:**
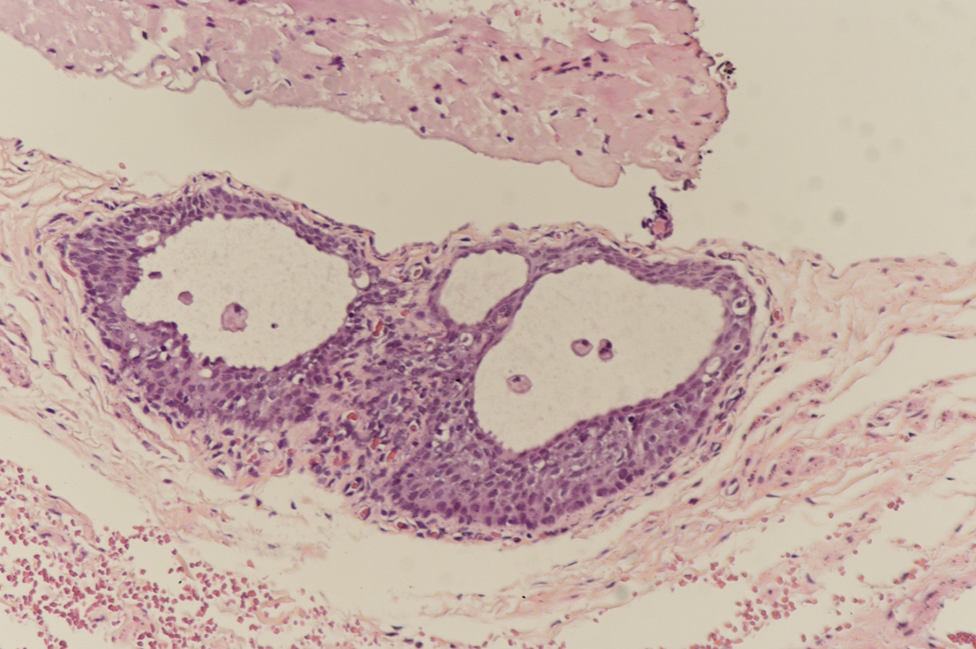
In the left gonad, microscopic showing fibers and two glandular tubular structures were observed (high magnification).

After admission, and with the aid of relevant examinations, the patient was diagnosed with TS, sex chromosome abnormalities, a right retroperitoneal tumor, and an adrenal mass. Cystoscopy with laparoscopic exploration, gonadal exploration, and posterior laparoscopic resection of the right adrenal mass under general anesthesia was conducted in the hospital’s Department of Urology on August 10, 2021. During the exploration, the bladder was seen to be normal, a uterus-like structure was found in the pelvic cavity, and the size of the cavity was identified as small (as expected in a patient of this age). A fallopian tube-like structure and cord-like gonads were visible on the left and right sides. Pathological investigation of a frozen intraoperative specimen found fibers, blood vessels, nerve fibers, and a glandular tubular structure in the left gonad (Figure 2) and blood vessels, nerve fibers, a seminiferous duct, a glandular tubular structure, and a small nest of cytoplasmic cells in the right gonad. With the approval of the hospital’s ethics committee and the informed consent of the patient’s parents, the left and right cord-shaped gonads were removed.

**Figure 3:**
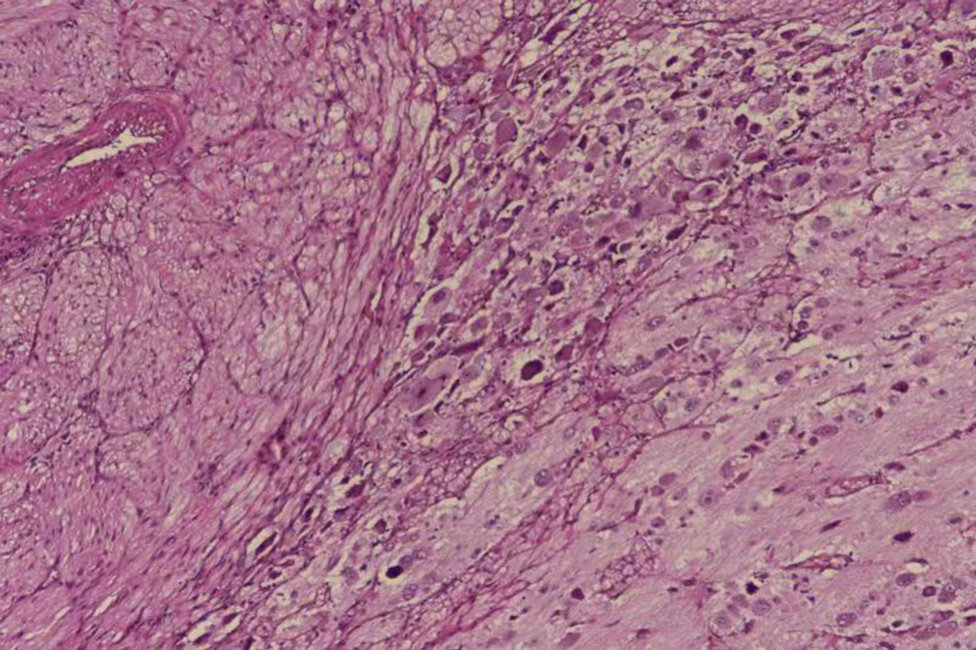
The right adrenal gland showed an abundant Schwann cell matrix scattered in neuroblastoma nests with different degrees of differentiation, and neuropils were visible in the nest. Schwann cell matrix >50%, with more calcification and lymphocytic foci (high magnification).

Postoperative pathology revealed left gonadal adenoblastoma, right gonadal stromal hyperplasia, and ganglion neuroblastoma (mixed type) in the right adrenal gland (Figure 3). The patient’s parents are currently considering whether to consent to the patient undergoing chemotherapy, and outpatient follow-up is being conducted in the hospital.

## 3 Discussion

One of the most common chromosomal abnormalities, TS, is also the only viable monomer syndrome in humans [2]. Typical clinical manifestations include hypoplasia of the secondary sex characteristics, primary amenorrhea, short stature, body deformity, and infertility. The condition can also be accompanied by a series of endocrine abnormalities [3], such as glucose metabolism disorder and thyroid disease.

To date, few studies have been published on the incidence of tumors in patients with TS, both in China and abroad. Hasle et al. [4] followed up 597 female patients with TS and found a total of 21 patients with tumors, an incidence of approximately 3.5%. Viuff et al. [5] found that the risk of benign central nervous system tumors, colon and rectal cancers, benign skin tumors, and skin cancers was increased by two to five times in patients with TS. Patients with TS with a 45, X/46, XX karyotype have also been found to have an increased risk of tongue cancer. In China, Lei et al. found that patients with TS of mosaic-type Y have an increased risk of gonad germ cell tumors [6], with a likelihood of approximately 5–20% and a reported rate of up to 30%. This may be related to the mutation of the Y chromosome gene (GBY), loss of female phenotype, and increased risk of gonadal development into gonadoblastoma in patients with gonadal hypoplasia. Therefore, for TS with Y chromosome mutation, bilateral gonadectomy is recommended to prevent the occurrence of gonadal tumors.

Neuroblastoma is the most common extracranial tumor in children, accounting for approximately 6–10% of tumors in childhood [7,8] and having a mortality rate of approximately 15%. It can originate in any nerve ridge of the sympathetic nervous system, most commonly the adrenal gland, and can also occur in the nerve tissues of the neck, chest, abdomen, and pelvis. The clinical presentations of patients with neuroblastoma vary with age and stage. The early symptoms in most children are not obvious, and only some patients show non-specific symptoms, such as anemia, fatigue, fever, and weight change. The size and location of primary and metastatic tumors determine the clinical manifestations [9].

Schoemaker et al. [10] found that, in addition to the increased risk of gonadoblastoma in patients with TS, there was also an increased risk of extragonadal tumors, such as meningioma, brain tumor, bladder cancer, melanoma, and uterine cancer. This might be related to the expression of a single allele of the tumor suppressor gene or DNA repair gene on the X chromosome of patients with TS. There have also been international reports on TS combined with hepatocellular carcinoma [11], papillary thyroid carcinoma [12], and neurogenic tumors [13] after treatment with recombinant human GH.

To date, there are nine foreign reports related to TS complicated with adrenal neuroblastoma but no relevant reports in China. This study reviewed and summarized the previously reported cases, and the details are shown in Table 1. A review of the relevant literature found that patients with TS have an increased likelihood of neuroblastoma and adrenal-related tumors and that the clinical manifestations are mostly atypical. Most of the cases reported were identified by chance during abdominal ultrasound or CT examinations, and the recurrence rate during follow-up was low. Susceptibility may be related to the loss of the X chromosome and changes in hormone levels in the body. Satgé et al. [13] found that the risk of neuroblastoma increased in patients with TS with the monomer karyotype and stated that the presence of genes on the X chromosome was related not only to the occurrence but also to the differentiation of neurogenic tumors. Neuroblastoma is derived from neural crest cells that generally express enzymes required for catecholamine metabolism. These enzymes produce vanillylmandelic acid (VMA) and homovanillic acid (HVA) after degrading norepinephrine, adrenaline, and dopamine. Detection of elevated levels of VMA and HVA in the serum and urine can therefore be adopted as a diagnostic basis. Approximately 75% of neuroblastoma patients have increased VMA and HVA levels [14]. In addition, neuron-specific enolase (NSE) and lactate dehydrogenase (LDH) have some significance in the diagnosis of neuroblastoma, and elevated NSE and LDH often predict a poor prognosis. Given the atypical clinical manifestations of neuroblastoma in patients with TS, screening catecholamine metabolites in the urine of patients with TS could help clinicians identify adrenal tumors early and avoid missed diagnosis and delayed treatment.

**Table 1:** Reported cases of gangliocytoma with TS

Case	Author	Publication time	Chromosomal karyotype	Age of diagnosis	Location and size of the tumor	The application of GH	Clinical manifestation	Follow-up after surgery
1	Miller et al. [15]	1968	45, X	4 years and 4 months	Bilateral adrenal gland	No	Hypertelorism, short neck, puffiness of dorsa of hands and feet since birth, mental retardation	No follow-up
2	Miller et al. [15]	1968	45, X	1 month	Bilateral adrenal gland	No	Cleft palate; hyperflexibility of joints; club-foot, bilateral; short, webbed neck; low-set ears; cerebral dysplasia of paraventricular regions with the absence of rt. choroid plexus	No follow-up
3	Wertelecki et al. [16]	1970	45, X	4 months	Bilateral adrenal gland	No	Not described	No follow-up
4	Matsuoka et al. [17]	1997	45, X	10 years and three months	Left adrenal gland, with a size of 6.5 cm × 6 cm × 5 cm	Yes (concurrence)	Neonatal echocardiography showed partial ectopic pulmonary vein reflux, limb edema, webbed neck, and microjaw deformity. At 6.8 years of age, the patient was 101.5 cm (−1.84 SD) tall and weighed 18.0 kg (−1.09 SD). The GH stimulation test showed a peak GH of 5.2 μg/L. GH therapy was started and 0.5 IU/kg of GH was added weekly. There was an average height increase of 5.1 cm within 3.5 years	No follow-up
5	Blatt et al. [18]	1997	46, XisoXq	4 years and 7 months	Left adrenal gland, with a size of 6 cm × 3 cm × 5 cm	Not specified	Diagnosed with epilepsy at 14 months and had a good response to ethosuximide treatment	No recurrence after 3 years of follow-up
6	Sasaki et al. [19]	2000	45, X	6 years old	Left adrenal gland	No	Limb edema, neck and jaw deformity	No follow-up
7	Stasik et al. [20]	2005	Not specified	31 years old	Left adrenal gland, with a size of 2 cm × 1 cm × 5 cm	Yes (before)	Webbed neck and short stature, Hashimoto's thyroiditis, unexplained elevated transaminase. GH treatment started at age 15 and for approximately 3 years	No follow-up
8	Kamoun et al. [21]	2010	Not specified	15 years old	Left adrenal gland, with a size of 6.5 × 5 × 3 cm. The weight was 70 g	No	Lymphedema of feet at birth, spontaneously resolute, and surgical repair of aortic arch abnormalities at the age of 6 years old. Height of 143 cm (−4 SD), weight of 45 kg (−1.5 SD), body mass index of 23.3 kg/m^2^ (between 90 and 97 percentile), blood pressure of 120/60 mm Hg, webbed neck, cubitus valgus, shortening of fourth metacarpal bones, and puberty retardation (Tanner stage 3)	No recurrence had developed until 9Months of follow-up
9	Wikiera et al. [22]	2013	45, X	11.5 years old	Right adrenal gland	Seven months after the administration of GH	Hypertelorism, short neck, puffiness of dorsa of hands, short stature	No follow-up

## 4 Conclusion

Children with TS have an increased likelihood of developing neuroblastoma and adrenal-related tumors, and changes in adrenal hormone levels and clinical manifestations are often not obvious when combined with adrenal-related tumors. Therefore, screening for adrenal tumors is recommended for patients with TS before the initiation of GH treatment to avoid missed diagnosis and delayed treatment.
